# Injection Molded Capsules for Colon Delivery Combining Time-Controlled and Enzyme-Triggered Approaches

**DOI:** 10.3390/ijms21061917

**Published:** 2020-03-11

**Authors:** Federica Casati, Alice Melocchi, Saliha Moutaharrik, Marco Uboldi, Anastasia Foppoli, Alessandra Maroni, Lucia Zema, Christel Neut, Florence Siepmann, Juergen Siepmann, Andrea Gazzaniga

**Affiliations:** 1 Sezione di Tecnologia e Legislazione Farmaceutiche “Maria Edvige Sangalli”, Dipartimento di Scienze Farmaceutiche, Università degli Studi di Milano, 20133 Milano, Italy; fcasati@continuuspharma.com (F.C.); alice.melocchi@unimi.it (A.M.); saliha.moutaharrik@unimi.it (S.M.); marco.uboldi@unimi.it (M.U.); anastasia.foppoli@unimi.it (A.F.); alessandra.maroni@unimi.it (A.M.); andrea.gazzaniga@unimi.it (A.G.); 2 IMA S.p.a., Ozzana dell’Emilia, 40064 Bologna, Italy; 3 University of Lille, Inserm, CHU Lille, UMR1286, F-59000 Lille, France; christel.neut@univ-lille.fr; 4 Université of Lille, Inserm, CHU Lille, U1008, F-59000 Lille, France; florence.siepmann@univ-lille.fr (F.S.); juergen.siepmann@univ-lille.fr (J.S.)

**Keywords:** capsules, colon delivery, injection molding, swellable/soluble hydrophilic polymer, bacteria-sensitive polymer

## Abstract

A new type of colon targeting system is presented, combining time-controlled and enzyme-triggered approaches. Empty capsule shells were prepared by injection molding of blends of a high-amylose starch and hydroxypropyl methylcellulose (HPMC) of different chain lengths. The dissolution/erosion of the HPMC network assures a time-controlled drug release, i.e., drug release starts upon sufficient shell swelling/dissolution/erosion. In addition, the presence of high-amylose starch ensures enzyme-triggered drug release. Once the colon is reached, the local highly concentrated bacterial enzymes effectively degrade this polysaccharide, resulting in accelerated drug release. Importantly, the concentration of bacterial enzymes is much lower in the upper gastrointestinal tract, thus enabling site-specific drug delivery. The proposed capsules were filled with acetaminophen and exposed to several aqueous media, simulating the contents of the gastrointestinal tract using different experimental setups. Importantly, drug release was pulsatile and occurred much faster in the presence of fecal samples from patients. The respective lag times were reduced and the release rates increased once the drug started to be released. It can be expected that variations in the device design (e.g., polymer blend ratio, capsule shell geometry and thickness) allow for a large variety of possible colon targeting release profiles.

## 1. Introduction

In the field of oral modified-release, great efforts have been made since the 1990s to develop drug delivery systems (DDSs) able to release the conveyed drug to specific regions of the gastrointestinal tract [1,2,3,4,5,6,7]. In particular, the colon drew considerable attention as a target site for the treatment of local disorders, such as inflammatory bowel diseases (e.g., ulcerative colitis and Crohn’s disease) and irritable bowel syndrome, as well as for the prevention of colorectal adenocarcinoma [8,9,10]. Moreover, the colonic region was investigated as a possible gateway to the systemic circulation, for instance to enhance the oral bioavailability of peptide and protein drugs [11,12,13]. Several strategies were proposed to achieve colon-targeted systems, based on the exploitation of one or more of the physiological features of the intestine [1].

Drug release into the large bowel was carried out using enzymatically degradable coatings, triggered by the in situ activity of the microbiota [14,15]. Recent in vivo studies carried out in healthy volunteers showed that these systems could be more reliable than pH-dependent ones [16,17]. The latter involve coatings dissolving above a pH value in the range of 5–7. Since the dissolution of the coating takes some time (depending on its thickness and exact pH threshold value), these systems should prevent the release of the incorporated drugs in the stomach and proximal bowel and release the drug in the subsequent parts of the gastrointestinal tract. However, both premature drug release in the small intestine, as well as no drug release at all, have been reported as potential failures [18]. This can be attributed to the significant variability of the pH of the contents of the different segments of the gastrointestinal tract. In this respect, attempts have been made to avoid release failure, due to insufficient exposure of the enteric-soluble layer to fluid with appropriate pH, by adding superdisintegrants to such coatings [19].

Polysaccharides such as chitosan, guar gum, pectin and chondroitin sulphate were used as release-triggering components, to be specifically degraded by resident bacteria [20,21,22]. Starch derivatives, especially those modified to resist pancreatic amylases, were also proposed [23,24,25]. However, the effectiveness of the above-mentioned materials as “colon carriers” is hampered by their hydrophilicity/solubility, possibly leading to the failure of the barrier properties before the colon is reached.

Time-dependent colonic DDSs were designed to undergo a silent phase of predetermined duration, after which drug release takes place [26,27]. The lag phase is intended to correspond to the relatively consistent small intestinal transit time, which is known to last about 3 h on average, with relatively limited variability, despite differences in the size and density of the administered dosage forms and feeding state of subjects [28]. In these cases, an external enteric film coating is needed to avoid variability of drug release due to unpredictable gastric emptying. Over the years, a variety of time-dependent reservoir systems have been described. Often, drug-containing cores are coated with layers based on hydrophilic cellulose ethers, such as hydroxypropyl methylcellulose (HPMC), hydroxypropyl cellulose (HPC), hydroxyethyl cellulose and calcium or sodium carboxymethylcellulose, in view of their well-established safety, versatility and broad availability [29,30]. When exposed to aqueous media, these polymers undergo more or less rapid swelling, dissolution and/or erosion, thus resulting in a deferred onset of drug release. The duration of the lag phase can be programmed by selecting the appropriate type of swellable/soluble hydrophilic polymer, its molecular weight (i.e., viscosity grade) and the thickness of the applied layer. In the case of barriers based on HPMC, different chain lengths and several coating techniques were studied [4,31]. More recently, time-dependent reservoir systems in the form of capsules were proposed [32,33,34]. Particularly, HPC was the first thermoplastic cellulosic derivative employed for the fabrication of capsule caps and bodies via injection molding (IM) to convey drug-containing preparations [35,36]. The capsules registered under the name of Chronocap™ showed the ability to release their contents after a predetermined lag phase, tunable according to the molecular weight of the selected HPC and the thickness of the molded shell. Moreover, when used as the substrate for enteric coating, the Chronocap™ system met the compendial gastroresistance requirements, while maintaining the subsequent pulsatile release performance, thus proving suitable for time-dependent colon delivery [37]. Because modulation of the lag phase could be a long and costly task, involving the development of new formulations, molds and molding processes, the prototyping ability of 3D printing by fused deposition modeling versus IM was recently investigated [38,39,40].

As a further development in the field of colon targeting, a novel path that combines microbiota and pH-dependent approaches was described [41]. For this purpose, a pH-sensitive polymeric film containing pancreatic amylase-resistant starch (added as enzyme-degradable pore former) was applied to drug-loaded tablets. The obtained reservoir systems were studied in healthy volunteers to assess the site of disintegration using γ-scintigraphy. Disintegration of the dosage forms was consistently observed at the ileocaecal junction or in the large intestine. Despite the increased chances of avoiding failure in drug release, issues associated with a premature release were reported.

Given these premises, the aim of the study was to evaluate the suitability of IM for the manufacturing of a novel type of capsules for colonic drug delivery, combining swellable/soluble hydrophilic polymers and polysaccharides degraded by bacterial enzymes. The former component may provide a lag phase due to a limited permeability and sufficient mechanical stability of the swollen gel in the upper gastrointestinal tract, thus allowing the device to transit through the small intestine without breaking up. On the other hand, the enzyme-degradable polymer, due to its selective degradation in the colon, should speed up the in situ breakup of the capsule shell undergoing hydration/dissolution. The combination of these two mechanisms, a “mixed time-controlled and enzyme-triggered approach” for colon targeting, could in principle help circumventing inherent limitations and variability issues related to single-trigger systems, thereby improving the site selectivity of drug release. Indeed, the bacteria-sensitive component may enable prompt and complete release, even in the case of systems with a lag phase that turns out longer than the real small intestine transit time, thus helping to prevent drug release that is too late (and cases with no drug release at all).

## 2. Results and Discussion

### 2.1. Hot-Processability of the Starting Materials

Pharmaceutical-grade polymers were studied, which should, in principle, be suitable for the development of reservoir systems allowing for colon delivery, relying on either the time-dependent or the enzyme-triggered approach.

Among the possible swellable/soluble hydrophilic polymers currently used for the formulation of pulsatile-release systems, HPC, HPMC and poly (vinyl alcohol) (PVA) were selected, based on experience previously gained with respect to their hot-processability [42,43,44]. Due to the fact that HPC, alone and in admixture with a plasticizer (i.e., polyethylene glycol 1500, PEG 1500), was studied in terms of thermo-mechanical properties and melt-viscosity for the development of a dedicated capsule-shaped mold, such a material was taken as a reference [36]. Moreover, a pilot industrial plant for HPC extrusion provided with online control systems was developed and the compliance of elemental and microbiological contaminants, as well as of by-products, with internal specifications was assessed [40]. This would be of crucial importance in view of the need to demonstrate the safety of the capsule under development. HPMC-based coating barriers applied by different techniques onto drug-containing cores were already demonstrated, to be able to provide reproducible lag phases prior to drug release [45]. Novel grades of HPMC with improved thermal properties (i.e., AffiniSol™) were recently proposed and mainly employed for the preparation of solid dispersions [46,47,48]. These grades are expected to maintain the crystallization-inhibiting properties of standard HPMCs, but can be extruded over a wider range of temperatures than the latter. In order to allow for different release profiles, AffiniSol™ with two different molecular weights was studied (i.e., HPMC 15LV and HPMC 4M). For the same reason, two PVA grades were selected (i.e., PVA 05 and PVA 40).

On the other hand, high amylose maize starch (i.e., Amylo^®^ N-460, AMY) and modified hydroxypropyl pea starch (i.e., Lycoat^®^ RS780, LYC) were deemed interesting based on reports in the literature concerning their enzymatic biodegradability [49,50]. In addition, starch itself and a few of its derivatives were successfully processed by hot melt extrusion (HME) and IM [51,52]. In these cases, mixtures of water and glycerol (GLY) were used as plasticizers.

Based on prior art knowledge, a preliminary HME study was performed, comparing different formulations (i.e., varying in the type and amount of plasticizer) and processing parameters. Suitable conditions were identified by progressively adjusting the temperature and screw speed, recording the extrusion stress and qualitatively evaluating the obtained products (e.g., transparency, homogeneity, presence of signs highlighting breaking of the flow, resistance to manual breaking) (Table 1).

Like the reference HPC, both investigated grades of HPMC could be processed at a temperature ≤ 160 °C when adding 10% PEG 1500. Despite the good characteristics of the PVA-based extrudates in terms of homogeneity, transparency and resistance to manual breaking, both grades of this polymer required relatively high processing temperatures, independent of the amount of added plasticizer (GLY). According to the literature, HME of starch derivatives generally requires the addition of a mixture of plasticizers, often water and GLY. The latter promote the formation of thermoplastic starch under heat and shear stresses [53,54]. Such a phenomenon, i.e., gelatinization, refers to the disruption of the granule structure of the starch, with loss of order and crystallinity, following the reduction of the hydrogen bonds between molecules with ease of mutual movements. Some of the main disadvantages associated with thermoplastic starch include retrogradation and unsatisfactory mechanical properties (i.e., fragility). Importantly, the presence of GLY can help in avoiding these drawbacks, and GLY does not easily evaporate during processing. When AMY was extruded, higher amounts of plasticizers and higher screw speed were required compared to LYC to obtain extrudates with similar, desired characteristics.

The first IM trials were used to manufacture polymeric disks. The process involves the flowing of a melt in all directions from a central injection point to the equidistant walls of the mold. These disk-shaped devices were shown to be very useful for the evaluation of the processability of polymeric formulations by IM [33]. In particular, the diameter of the disks with respect to that of the mold, and the need for intervention during the ejection process, as a consequence of the adhesion of the object to the mold, were the parameters used to define a “qualitative processability scale” describing a single batch, i.e., the molding of 30 consecutive items. Moreover, the disks could be used to investigate the mechanism of interaction with aqueous fluids (i.e., water uptake and dissolution/erosion rate) of the polymeric formulations and, using them to close the donor compartment of modified permeability cells, the relevant release-controlling potential could be studied in a quantitative manner, i.e., measuring the time needed to rupture the sample. In view of the final goal, i.e., the development of a delivery platform combining time-dependent and microbiological approaches for colon targeting, not only formulations based on single polymers (i.e., either swellable/soluble hydrophilic polymers or starch derivatives), but also combinations (1:1 weight: weight ratio) of two polymeric formulations, composed of a swellable/soluble hydrophilic polymer and of a bacterial degradation-sensitive polymer, respectively, were taken into account. Preliminary studies relevant to disk manufacturing allowed the setup of adequate process parameters, i.e., temperature and injection pressure, time as well as rate. These are reported in Table 2 together with processability scores for each polymeric formulation employed and pictures of the best products that were obtained.

Since the injection cycle was shorter than the HME process and the temperature could be progressively increased along the different sections of the press, it was possible to set higher temperatures for IM than for HME, especially in the case of the investigated starch derivatives, without impacting the product quality. In particular, temperatures in the metering zone and in the nozzle were increased in order to achieve a viscosity of the melt, enabling the complete filling of the mold cavity. AMY was used to evaluate whether an optional HME of the polymeric formulation prior to IM affected the thermoplastic behavior of the starch derivatives. The plasticized AMY formulation was directly introduced into the micromolding equipment. Alternatively, the equipment was fed with extruded pellets, prepared with the same plasticized AMY formulation. The water content of the extruded pellets turned out to be critical for the subsequent IM process. In fact, optimal operating IM parameters set for the polymeric formulation and the pellets were the same, except for the temperature, which needed to be slightly increased for the pellets. This could be attributed to the lower water content of the pellets, some of the water probably being lost during the HME process. Irrespective of an optional HME step, AMY showed a good moldability, even if disks needed to be manually removed from the ejector. In contrast, the processability of the LYC formulation was not satisfying, with recurrent blockage of the apparatus and sporadic achievements of entire and non-deformed disks. This behavior was not altered when adding HME step and feeding the micromolding equipment with the obtained pellets. Among the investigated formulations based on swellable/soluble hydrophilic polymers, only PVA (especially the higher molecular weight grade PVA 40), showed poor processability, even when increasing the temperatures and the amounts of plasticizer.

The plasticization conditions found suitable for molding the formulations based on single polymers turned out to be also appropriate for the respective blends. Moreover, the processing temperatures and the final characteristics of the obtained products were found to be similar for formulations based on the respective single polymers and blends. In particular, processing was especially challenging in the case of blends, including at least one polymeric formulation, which showed difficulties during molding as single polymer formulation. Specifically, no disks were obtained that could be tested in the modified permeability cells that were based on single polymer formulations or blends containing LYC and PVA 40.

### 2.2. Performance of Molded Disks

#### 2.2.1. Interaction with Aqueous Fluids

In order to assess the potential of the selected formulations to act as release-controlling barriers, molded disks were preliminarily tested for interaction with aqueous fluids.

**Figure 1:**
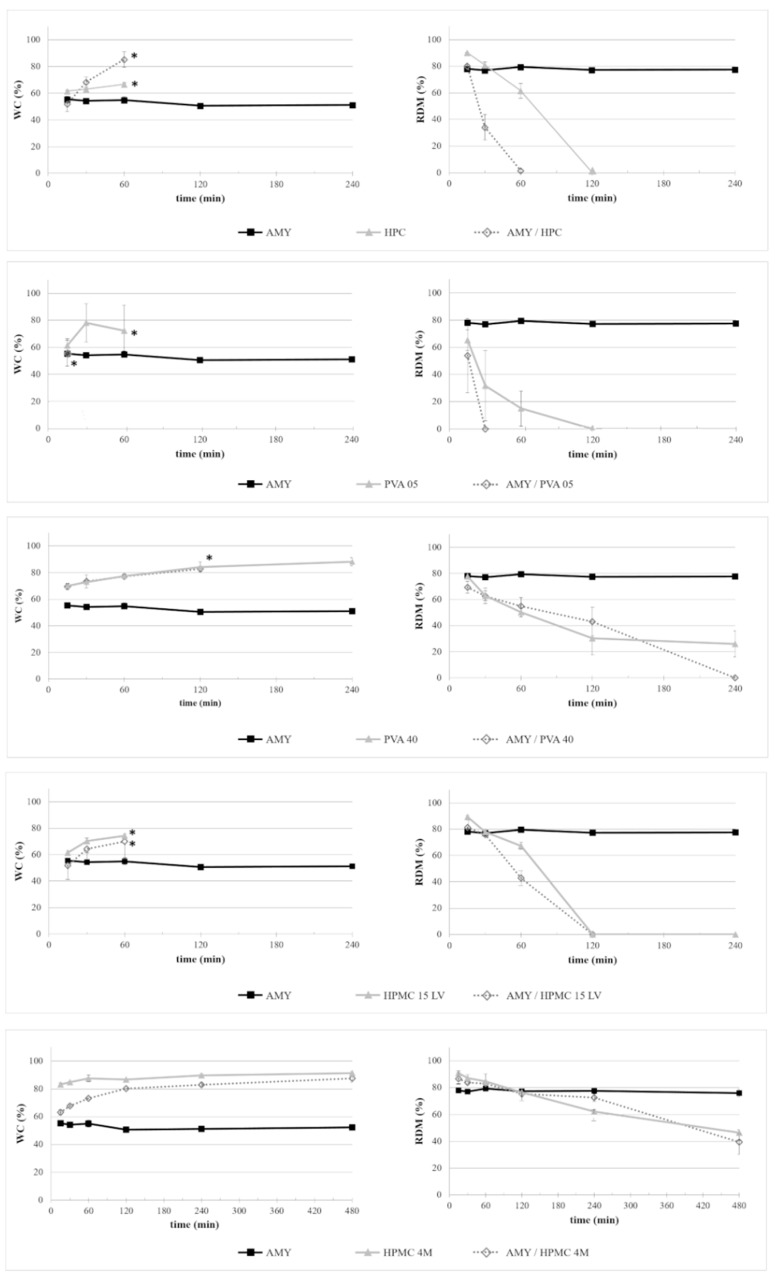
Dynamic changes in WC and RDM of disks based on AMY or blends of AMY with a swellable/soluble polymer hydrophilic upon exposure to phosphate buffer pH 6.8. For reasons of comparison, the behavior of disks based on the respective swellable/soluble hydrophilic polymer are also shown. * in the WC profiles marks the last recorded data before the complete dissolution of samples.

**Figure 2:**
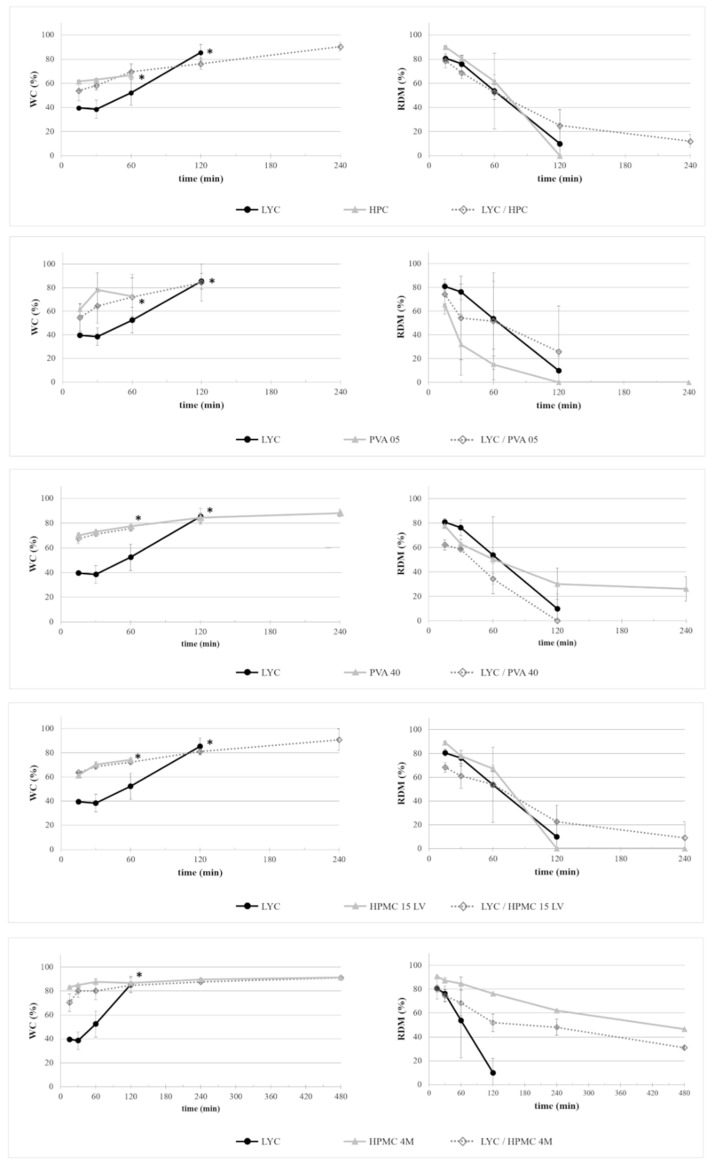
Dynamic changes in WC and RDM of disks based on LYC or blends of LYC with a swellable/soluble hydrophilic polymer upon exposure to phosphate buffer pH 6.8. For reasons of comparison, the behavior of disks based on the respective swellable/soluble hydrophilic polymer are also shown. * in the WC profiles marks the last recorded data before the complete dissolution of samples.

The dynamic changes in the water content (WC) and residual dry mass (RDM) of disks based on AMY or LYC, optionally blended with different swellable/soluble hydrophilic polymers upon exposure to phosphate buffer pH 6.8, are shown over 8 h in Figure 1; Figure 2, respectively. For reasons of comparison, the behavior of the respective disks based on the swellable/soluble hydrophilic polymers are also shown.

**Figure 3:**
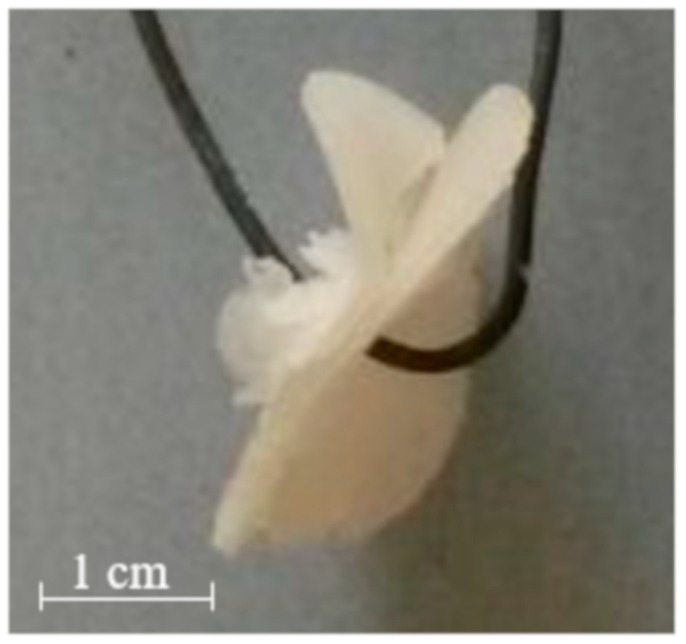
Picture of an AMY-based disk after 5h exposure to phosphate buffer pH 6.8.

AMY-based disks showed a moderate uptake of water during the first minutes of testing, without any evidence for major increase in volume. The water content reached was about constant over time. Accordingly, after an initial moderate dry mass loss, no further reduction was observed. The RDM value remained relatively high (≥ 80%) until the end of the experiment. This is likely due to the high amount of amylose, providing resistance to dissolution/erosion. However, after a few hours of testing, a tendency of AMY-based disks to exfoliation was noticed (Figure 3). This phenomenon also affected the barrier performance, leading to the rupture of the disks in less than 30 min (the earlier rupture in this case can probably be attributed to the more stressful hydrodynamic conditions encountered during this type of experiment). Disks based on AMY, but prepared via an additional HME step, behaved similarly with respect to their water uptake, mass loss and exfoliation behavior. On this basis, the extrusion step was considered not to be essential for the subsequent IM process. Therefore, the direct molding of powders was carried out during all further experiments.

Disks based on HPC, PVAs and HPMCs showed generally higher water uptake rates and extents and more pronounced and more rapid dry mass loss kinetics upon exposure to phosphate buffer pH 6.8 than disks based on AMY (Figure 1; Figure 2). As expected, the swelling ability and dissolution/erosion rate were related to the type and molecular weight of the polymer. HPMC 4M- and PVA 40-based samples, in particular, achieved a water content above 85% and maintained it over 8 h, accompanied by a significant mass loss. However, they did not completely dissolve in the observation period (8 h), maintaining RDM values of approximately 30% and 50% for PVA 40- and HPMC 4M-based items, respectively. The dissolution/erosion process of disks based on HPC and on the lower molecular weight grades of both PVA and HPMC was relatively fast and complete in about 2 h. These results were consistent with the observed rupture times of the disks tested using the modified permeability cells (i.e., HPMC 4M >> HMPC 15LV > PVA 05 ≈ HPC).

According to the combination of approaches to provide colon delivery as pursued in this study, the dissolution/erosion of the gel barrier is intended to be sufficiently slow to assure that the devices may pass intact through the small intestine and provide an appropriate lag time prior to drug release. This is why it was important to also investigate the behavior of disks based on the respective polymer blends. In the case of disks based either on HPC or PVA 05, the addition of AMY strongly reduced the time needed for complete dissolution, by 50% or even 75%. While a slight decrease in the water content was observed with discs containing HPMC of both molecular weights when adding AMY, no major impact on the respective dry mass loss was found. Thus, this type of polymer blend might be particularly promising in view of the desired behavior. No AMY/PVA 40-based disks could be withdrawn at the end of the test, even though the initial mass loss rate was similar to that of disks based on PVA 40 only. This might be attributable to the lack of integrity and low quality of the disks even before exposure to the phosphate buffer, which also made their testing in the barrier performance experiments impossible. The rupture times observed during the latter tests with the other types of disks confirmed the above described observation with respect to the water uptake and mass loss kinetics (i.e., AMY addition led to increased rupture times in the case of HPMC 15LV- and HPMC 4M-containing prototypes and decreased rupture times in the case of HPC- and PVA 05-containing disks). Interestingly, the exfoliation issues observed with disks based on AMY only were not encountered in any of the relevant blends.

Disks based on LYC showed a slower water uptake compared to AMY-based disks, but a complete mass loss in less than 3 h, which was expected considering the soluble nature of this polymer. When used in blends, LYC seemed not to accelerate the mass loss of disks with respect to prototypes based on the corresponding swellable/soluble hydrophilic polymer only, especially in the case of HPC, PVA 05 and HPMC 15LV. However, a considerable variability was observed with all LYC-containing samples, probably due to the above-mentioned poor quality of the molded items. Indeed, no entire disks for the measurements of rupture times were obtained. For these reasons, LYC was discarded from further experiments. On the other hand, blends of AMY with both grades of HPMC seemed to be the most promising candidates at this stage and were further investigated.

#### 2.2.2. Interactions with Culture Medium +/- Fecal Samples

**Figure 4:**
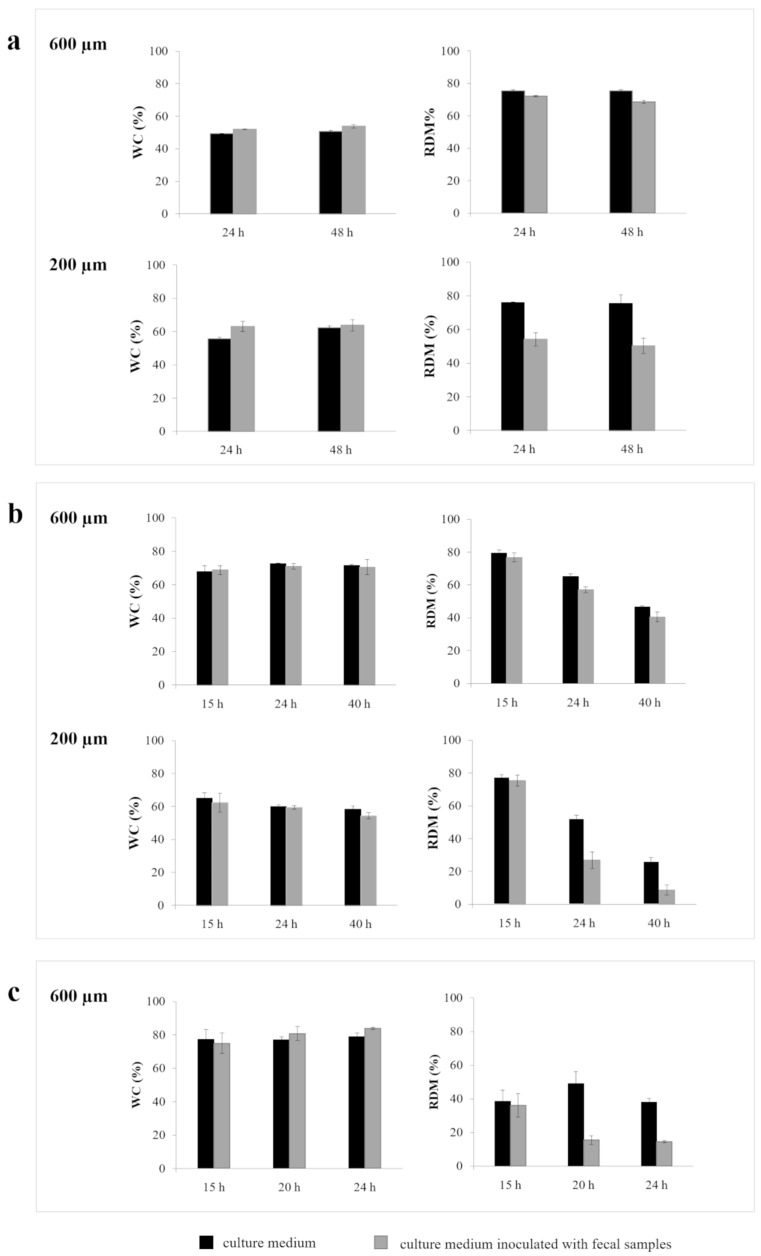
Dynamic changes in WC and RDM of: (**a**) 600 and 200 µm thick disks based on AMY, (**b**) 600 µm thick disks based on AMY/HPMC 4M and (**c**) 600 and 200 µm thick disks based on AMY/HPMC 15LV upon exposure to culture medium, or culture medium inoculated with fecal samples for different time periods.

According to the combined approach proposed here for colon delivery, the presence of the bacteria-sensitive component is intended to promote the fast opening of capsules once the site of interest is reached, due to specific degradation by enzymes secreted by the local microbiota. The use of biorelevant media, e.g., based on rat cecal contents and human fecal slurries, was already demonstrated to be a suitable strategy to evaluate the role of polysaccharide fermentation as a trigger for drug release [55]. Relying on the data described above obtained with phosphate buffer, disks based on AMY/HPMC 15LV and AMY/HPMC 4M were selected for further testing in aqueous fluids containing fecal bacteria. For reasons of comparison, disks based on AMY only were also studied. Please note that in addition to the 600 µm thick “standard” disks, 200 µm thick disks were also prepared by IM and studied. Figure 4 shows the dynamic changes in the WC and RDM of disks based on AMY, AMY/HPMC 15LV and AMY/HPMC 4M (200 or 600 µm thickness) upon exposure to culture medium free of fecal samples or inoculated with fecal samples, under anaerobic conditions.

As can be seen, disks based only on AMY confirmed their ability to take up and maintain a roughly constant water content during at least two days, irrespective of their thickness and the presence/absence of fecal samples. All the prototypes tested in the culture medium free of feces showed a mass loss of approximately 20% in the first 24 h, with no further changes in the rest of the experiment. This could be explained by the exfoliation behavior already observed in phosphate buffer for the same type of disks. However, when they were in contact with the fluids enriched with feces, a reduction in RDM values was observed, which was more evident with the thinnest barriers (i.e., mass loss about 50%). Such results indicate that colonic bacteria effectively cause the fermentation of the investigated starch derivative upon molding. The addition of swellable/soluble HPMC led to increased WC and accelerated mass loss, as expected (Figure 4b,c versus 4a). This is due to the hydrophilic nature and water solubility of the HPMC. Please note that in the case of the shorter chain HPMC, earlier sampling time points were used (i.e., 15 and 20 h). The presence/absence of fecal samples only slightly affected the measured dry mass loss behavior of the investigated 600 µm thick disks based on AMY/HPMC 4M under the given conditions. However, thinner disks (200 µm) showed a much more pronounced mass loss in the presence of fecal bacteria (Figure 4b). This is likely due to the fact that enzymatic degradation becomes more easily visible in the case of thinner samples (with a lower staring mass). However, in the case of injection molded AMY/HPMC 15LV disks that were 600 µm thick, a clear impact of the presence of fecal bacteria on the mass loss kinetics was visible (Figure 4c), indicating the sensitivity of these systems towards bacterial enzymes. The difference between HPMC 4M and HPMC 15LV containing samples can again be attributed to the different chain lengths of these polymers (please see above). In brief, HPMC 15LV leads to faster dissolution/erosion, thus favoring the attack of bacterial enzymes.

### 2.3. Manufacturing of and Drug Release from Capsules

In view of the promising results in terms of sensitivity to the colonic bacteria degradation observed with AMY in admixture with HPMC of different molecular weight, empty capsule shells with a nominal wall thickness of 600 µm based on these polymer blends were prepared by IM. Table 3 shows the final processing conditions and pictures of the respective capsules. For reasons of comparison, capsule caps and bodies based on single plasticized polymers are also shown. Please note that the processing conditions are slightly different from those applied for the manufacturing of the corresponding disks, because of the differences in the mold geometry and dimensions (e.g., halved thickness in the overlapping area between the cap and the body). For instance, the temperatures had to be increased in the hot runner to ensure a proper flow of the melt and allow its progression into the cavity. Overall, the IM process turned out to be slightly more challenging compared to the fabrication of the above described disks. Nevertheless, it was possible to obtain capsule shells with the desired physico-technological characteristics (i.e., reproducible weight and thickness, body and cap details enabling appropriate matching and leading to a seal closure of the capsules).

Filled and assembled capsules were tested in a basket-rack assembly of a modified disintegration apparatus (as reported in 3.2.6). The latter had been demonstrated to be adequate for the evaluation of reservoir systems based on swellable/soluble hydrophilic polymers, allowing the improvement of the reproducibility of release measurements and avoiding sticking, as well as floating issues [35,36].

**Figure 5:**
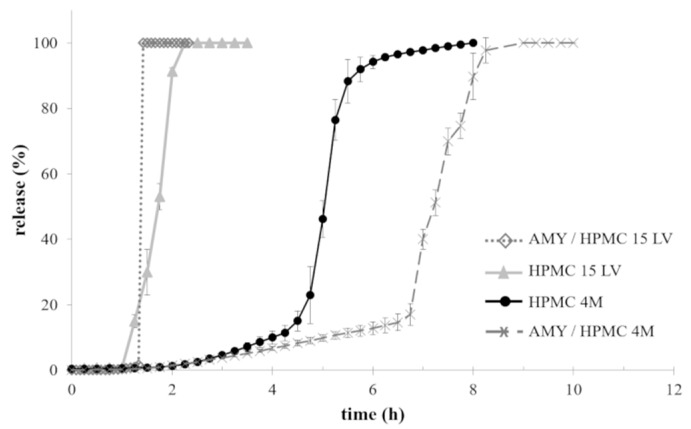
Acetaminophen release from capsules of different composition in phosphate buffer pH 6.8 using a modified disintegration apparatus.

**Figure 6:**
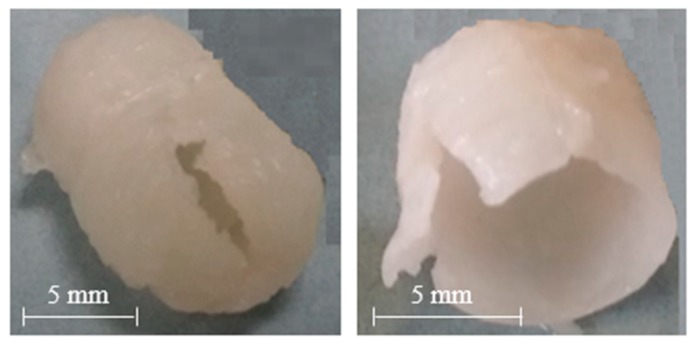
Pictures of capsules based on AMY only after 30 min exposure to phosphate buffer pH 6.8 in a modified disintegration apparatus.

Figure 5 illustrates the observed release kinetics of acetaminophen from capsules based on AMY/HPMV 15LV or AMY/HPMC 4M blends. For reasons of comparison, drug release from capsules based on HPMC 4M or HPMC 15LV is also shown. Note that the investigated capsules based on AMY only showed poor mechanical resistance under the given conditions (Figure 6), resulting in immediate release (data not shown). This was attributed to the above-described exfoliation behavior. The release performance was consistent with that of starch-based molded capsules registered under the trade name Capill^®^ [56,57,58]. On the other hand, HPMC-based capsules exhibited a pulsatile release profile, characterized by lag phases of different duration prior to release (Figure 5). These differences may be attributed to the characteristics of the swollen system (e.g., gel strength). The slower erosion/dissolution of the gel barrier based on HPMC 4M compared to HPMC 15LV was also reflected in a different time for complete release (i.e., 3 times longer than that of HPMC 15LV-based capsules). The addition of AMY to HPMC 15LV based capsules did not substantially alter the overall pulsatile release kinetics of these systems, which is consistent with the dry mass loss kinetics of the respective disks (please see above). Importantly, the addition of AMY significantly prolonged the lag time observed with HPMC 4M-based capsules (Figure 5). This illustrates the ability of AMY to slow down the hydration and dissolution/erosion of the HPMC gel.

**Figure 7:**
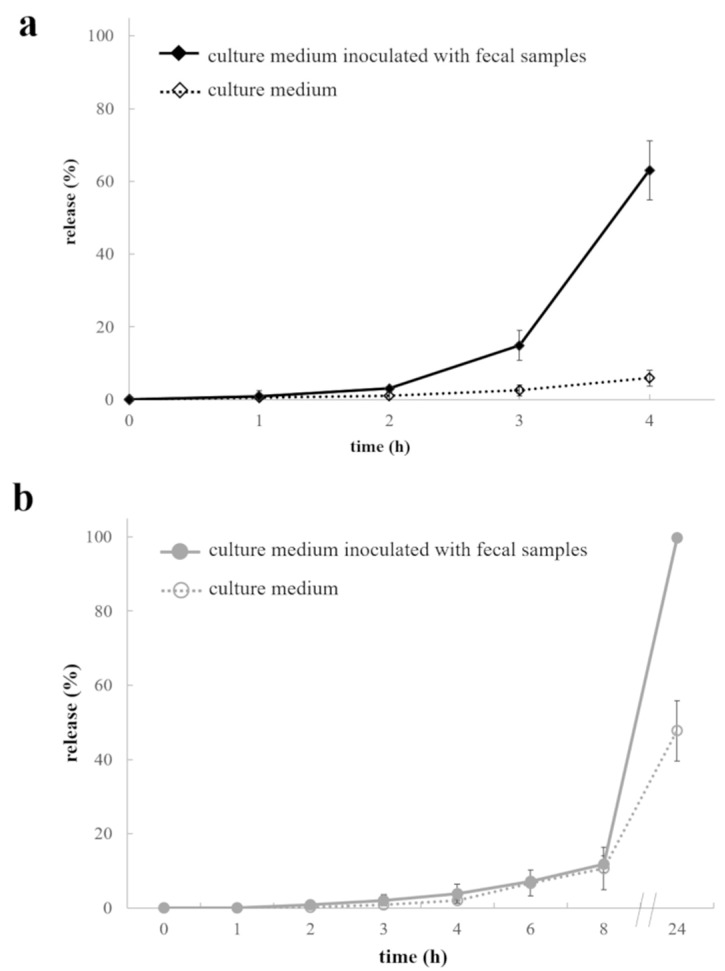
Acetaminophen release from capsules based on (**a**) AMY/HPMC 15LV and (**b**) AMY/HPMC 4M, upon exposure to culture medium free of bacteria, and culture medium inoculated with fecal samples.

To evaluate the possible role of AMY in the capsule shell as an enzyme-trigger, AMY/HPMC 15LV and AMY/HPMC 4M capsules were filled with acetaminophen and exposed to culture medium free of fecal samples, and culture medium inoculated with fecal samples from patients suffering from inflammatory bowel diseases (Figure 7).

As can be seen, drug release was substantially faster from the investigated capsules in the presence of fecal samples. Such a finding clearly validates the intended approach of enzyme-triggered drug release allowing for colon targeting. This was true for both grades of HPMC 4M and 15LV. The acetaminophen release was faster from AMY/HPMC 15LV based capsules compared to AMY/HPMC 4M based ones in the presence and absence of fecal samples. This can again be attributed to the difference in polymer molecular weight, as discussed above. Please note that the lag times for drug release were prolonged compared to the results observed using the modified disintegration apparatus (Figure 5), also in the absence of fecal bacteria. This can be attributed to the different mechanical stresses encountered in the two experimental setups during drug release (the difference between phosphate buffer pH 6.8 and the culture medium probably only plays a minor role).

## 3. Materials and Methods

### 3.1. Materials

Swellable/soluble hydrophilic polymers: HPMC (AffiniSol™ 15LV and 4M grades; Dow Chemical, Pittsburg, CA, USA; HPMC 15LV and HPMC 4M, respectively); HPC (Klucel^®^ LF, Ashland, Chatham, NJ, USA); PVA (Gohsenol™ EG 05P and EG 40P, Nippon Gohsei, Tokyo, J; PVA 05 and PVA 40, respectively).

Polysaccharides sensitive to bacterial degradation: high-amylose maize starch (Amylo^®^ N-460, Roquette Pharma, Souvigné, France; AMY); hydroxypropyl modified pea starch, (Lycoat^®^ RS780, Roquette Pharma, Souvigné, France; LYC).

Plasticizers: PEG 1500 (Clariant Masterbatches, Milan, Italy); GLY (Pharmagel, Milan, Italy).

Tracers: blue dye-containing preparation (Kollicoat^®^ IR Brilliant Blue, BASF, Ludwigshafen, D, Germany); acetaminophen (Rhodia, Milan, Italy).

### 3.2. Methods

#### 3.2.1. Preparation of Polymeric Formulations

Formulations based on a single polymer:

The polymer (AMY, LYC, HPC, HMPCs or PVAs) was kept in an oven at 40 °C for 24 h prior to placing it into a mortar. The plasticizer was manually added under continuous mixing with a pestle. The amount of plasticizer is expressed as percentage “weight by weight”, based on the dry polymer (=100%).

Formulations based on polymeric blends:

First, the single polymers were plasticized as described above: one swellable/soluble hydrophilic polymer and one polysaccharide sensitive to bacterial degradation. Then, the two plasticized polymers were manually mixed in a mortar in a 1:1 weight: weight ratio.

#### 3.2.2. HME

HME was performed using a twin-screw extruder (HAAKE^™^ MiniLab II, Thermo Scientific^™^, Bannockburn, IL, USA), equipped with two conical counter-rotating screws (diameter 5/14 mm, length 109.5 mm). Polymeric formulations were manually loaded into the barrel and extruded through a rectangular die (dimensions: 1 × 3 mm). The HME process parameters were set up and are reported in Section 2.1.

#### 3.2.3. IM

IM was performed using a bench-top micromolding machine (BabyPlast 6/10P; Cronoplast S.L., E; Rambaldi S.r.l., Lecco, Italy), equipped with: *i)* a disk-shaped mold (ø = 30 mm), with a central gate, allowing the variance of cavity thickness (nominal 200 and 600 μm) or *ii)* a mold with a hot-runner and two interchangeable inserts for the manufacturing of matching capsule caps and capsule bodies (600 µm nominal shell thickness). The polymeric formulations described in Section 3.2.1 were manually loaded into the plasticating unit of BabyPlast and fed into the injection chamber by a loading plunger. Two different and consecutive injection pressures (P_1_–P_2_), maintained for a selected time (t_1_–t_2_), were applied by a piston moving at two distinct rates (v_1_–v_2_; v is expressed as percentage of the maximum rate), in order to inject the polymeric melt into the mold cavity. For injection pressures and injection rates, minimum values were chosen and progressively increased until satisfactory products were obtained. In the case of AMY, disks were fabricated not only from the polymeric formulation described in Section 3.2.1, but also from pellets, which were manually cut from extrudates of the same composition and prepared as described in Section 3.2.2. The process parameters selected for the fabrication of disks and capsule shells are reported in Section 2.3.

#### 3.2.4. WC and RDM

Injection molded disks were characterized in terms of water uptake and mass loss upon exposure to different aqueous media, as described in the following.

Then, 600 μm thick disks (*n* = 9) were weighed (analytical balance BP211, Sartorius, Göttingen, D, Germany) to record the initial mass, and then immersed in 125 mL phosphate buffer pH = 6.8 (USP 42), and kept at 37 ± 0.5 °C under magnetic stirring (125 rpm). For the easy manual recovery of samples at the end of the test and prevention of damages, a customized metal support was used;

Accordingly, 200 μm and 600 μm thick disks (*n* = 9) were weighed (analytical balance BP211, Sartorius, Göttingen, D, Germany) to record the initial mass and then immersed in 100 mL of fluid after insertion into a 180 µm tubular mesh, closed with clips at both ends to facilitate manual recovery at the end of the test. The fluid was either free culture medium or culture medium inoculated with fecal samples of patients suffering from ulcerative colitis. Culture medium was prepared by dissolving 5 g tryptone, 3 g yeast extract, 2.5 g NaCl, 1.5 g beef extract and 0.3 g l-cysteine hydrochloride hydrate in 1 L of distilled water (pH = 7.0 ± 0.2) and subsequent sterilization in an autoclave (20 min at 115 °C). Fecal samples were diluted about 1:200 (final concentration = 0.0125% *w*/*v*) with cysteinated ringer solution, in order to have an initial concentration of about 7 log CFU/mL with only minor variation due to inherent individual differences. Furthermore, 2.5 mL of the fecal suspension was diluted with culture medium up to 100 mL. The systems were kept at 37 ± 0.5 °C under horizontal shaking (80 rpm; Stuart SSM1 Mini Orbital Shaker, VWR, Monroeville, PA, USA) and anaerobic conditions, as previously described in detail [49,50].

At pre-determined time points, samples were withdrawn, manually blotted to remove the excess of fluid, and weighed. The disks were then dried at 60 °C until constant weight. WC and RDM were calculated according to the following equations:

(1) $$WC\;\left(\%\right)=\left[\frac{\left(W_{m}-W_{d}\right)}{W_{m}}\right]\times 100$$

where W_m_ is the mass of the wet sample upon withdrawal at the sampling time point, and W_d_ is the mass of the respective sample after drying to constant weight;

(2) $$RDM\left(\%\right)=\left(1-\;\left[\frac{\left(W_{i}-W_{d}\right)}{W_{i}}\right]\right)\times 100$$

where W_i_ is the initial dry mass of the sample.

#### 3.2.5. Barrier Performance of Molded Disks

Molded disks (*n* = 6) were tested for barrier performance by using them to close the donor compartment of manually assembled cells, modified from the extraction cells used for the dissolution test of transdermal patches [33]. The donor compartment was filled with about 20 mg of Kollicoat^®^ IR brilliant blue as a tracer. The manually assembled cells were placed at the bottom of vessels of a USP 42 dissolution apparatus II (500 mL phosphate buffer pH 6.8 as the acceptor medium, 37 ± 0.5 °C, 100 rpm paddle rotating speed; Dissolution System 2100B, Distek, North Brunswick Township, NJ, USA). Barrier resistance was visually evaluated and quantified as the time to the appearance of a first tear on the disk (i.e., rupture time), highlighted by the coloring of the acceptor medium.

#### 3.2.6. In Vitro Release from Capsules

Capsule bodies (*n* = 6) were manually filled with approximately 50 mg (coefficient of variation < 2) acetaminophen and then closed with matching caps. Each assembled capsule was then inserted into a sinker. Drug release was measured as follows:

Using a modified three-position disintegration apparatus (Sotax, Lugano, CH): a single capsule was positioned in one of the 6 available tubes of each basket-rack assembly, that moved at 31 cycles/min in a vessel filled with 800 mL phosphate buffer pH 6.8, kept at 37 ± 0.5 °C. Fluid samples were automatically withdrawn at predetermined time points and assayed spectrophotometrically (λ = 248 nm; Lambda25, Perkin Elmer, UK).

Capsules were placed in closed flasks containing 100 mL culture medium (free of fecal samples), or 100 mL culture medium inoculated with fecal samples (1% *w*/*v*) from patients suffering from inflammatory bowel diseases (i.e., ulcerative colitis and Crohn’s disease) under anaerobic conditions. At predetermined time points, 2 mL fluid samples were withdrawn, centrifuged (13,000 rpm, 5 min) and filtered (0.22 µm), before being analyzed by HPLC (Thermo Fisher Scientific Ultimate 3000 Series, Boston, MA, USA). A Gemini^®^ 5 μm C18 110 Å, 150 × 4.6 mm column (Phenomenex, London, UK) was used. The mobile phase was a blend of: A) water adjusted to pH 2 with orthophosphoric acid, and B) acetonitrile. A linear gradient program was run as follows: 0–10 min: 5–20% B; 10–11 min, 20–5% B. The flow rate was 1 mL/min, and 10 μL samples were injected. Acetaminophen was detected spectrophotometrically (λ = 248 nm).

## 4. Conclusions

In the present work, the suitability of IM for the preparation of empty capsule shells as a platform for colon targeting was demonstrated, combining time-dependent and enzyme-triggered approaches. The composition of the systems (i.e., type and amount of plasticizer, types of polymers) and processing conditions were selected. By combining polymeric formulations based on AMY and HPMCs with different molecular weight, the possibility of modulating independent and complementary time-dependent and enzymatic-degradation mechanisms was demonstrated. This should allow circumventing issues related to single-trigger systems and improve the site selectivity of drug release. Furthermore, the selection of the swellable/soluble hydrophilic polymer might result in different release patterns and in vivo performance of the capsules, once the system has reached the colon. For example, the presence of a highly viscous gel characterized by a relatively slow dissolution/erosion rate, might either prevent or defer the exposure of AMY to the colonic fluids and therefore limit or slow down its degradation by the bacterial enzymes. Considering that the type and ratio of the two components in the blend can be adjusted, further possibilities of modulating the degradation behavior are likely, representing an advantageous aspect for the development of a novel colon delivery platform.

## Figures and Tables

**Table 1:** Composition, processing parameters and pictures of extrudates based on different polymers.

	HPC	HPMC 15LV	HPMC 4M	PVA 05	PVA 40	AMY	LYC
**Plasticizer (% w/w)**	PEG 1500 (10)	PEG 1500 (10)	PEG 1500 (10)	GLY (15)	GLY (15)	GLY (20) + water (15)	GLY (10) + water (5)
**Temperature (°C)**	150	155	160	170	190	105	100
**Screw Speed (rpm)**	50	80	80	50	30	75	50
**Torque (N·cm)**	25	60	80	40	95	60	80
	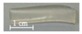	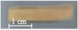	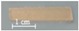	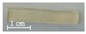	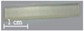	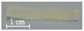	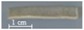

**Table 2:** Process parameters and pictures (the side of the squares in the background is 0.5 mm long) of molded disks, based on different polymeric formulations.

Plasticized Formulation Based on		Process Parameters						Processability *	
		Temperature (°C)			Injection Pressures, P_1_–P_2_ (bar)	Injection Times, t_1_–t_2_ (s)	Injection Rates, v_1_–v_2_ (%)		
		Compression Zone	Metering Zone	Nozzle					
**AMY**	Powder	110	115	130	70-60	0.8-0.3	40-20	-/+	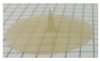
	Extruded pellets	110	120	140	70-60	0.8-0.3	40-20	-/+	
**LYC**		100	125	135	70-60	0.8-0.3	50-30	-	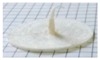
**HPC**		145	150	165	50-40	0.8-0.3	50-40	++	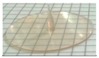
**HPMC 15LV**		155	165	175	50-30	0.8-0.3	30-10	++	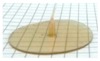
**HPMC 4M**		175	180	185	60-50	1.5-1.0	60-50	++	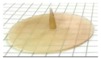
**PVA 05**		155	165	170	50-30	0.8-0.3	50-30	+	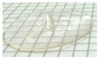
**PVA 40**		170	175	180	70-50	0.8-0.3	70-50	+/-	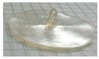
**AMY/HPC**		125	125	135	60-50	0.8-0.3	30-20	+	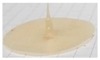
**AMY/HPMC 15LV**		125	125	135	30-20	2.0-1.5	30-10	+	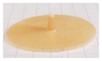
**AMY/HPMC 4M**		135	135	155	60-50	0.8-0.3	60-40	+	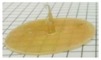
**AMY/PVA 05**		160	170	175	75-60	1.5-1.0	40-30	+/-	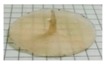
**AMY/PVA 40**		150	155	160	60-50	1.0-0.8	40-30	-	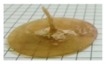
**LYC/HPC**		125	130	135	60-50	0.8-0.3	30-20	+/-	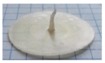
**LYC/HPMC 15LV**		125	135	145	80-60	0.8-0.3	60-50	+/-	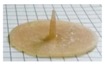
**LYC/HPMC 4M**		135	140	150	50-30	0.8-0.3	50-30	+/-	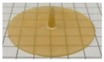
**LYC/PVA 05**		160	170	175	60-40	0.8-0.3	40-30	-	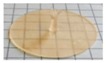
**LYC/PVA 40**		150	155	165	50-30	0.8-0.3	50-30	-	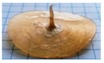

* −, mostly incomplete/broken/deformed disks; −/+, complete disks manually removed/extremely adhesive; +, complete disks, occasionally automatically ejected; ++, complete disks, automatically ejected.

**Table 3:** Process parameters and pictures of molded empty capsule caps and bodies based on different polymers.

Formulation	Process Parameters							Processability *	
	Temperature (°C)				Injection Pressures, P_1_–P_2_ (bar)	Injection Times, t_1_–t_2_ (s)	Injection Rates, v_1_–v_2_ (%)		
	Compression Zone	Metering Zone	Nozzle	Hot Runner					
**AMY**	110	115	130	135	70–60	0.8–0.3	40–20	+/−	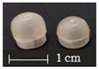
**HPMC 15LV**	165	170	180	190	30–10	0.5–0.3	30–10	+/−	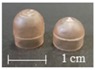
**HPMC 4M**	175	180	190	200	30–10	0.5–0.3	30–10	+/−	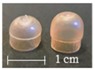
**AMY/HPMC 15LV**	120	125	135	145	30–20	0.8–0.3	30–10	+/−	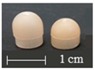
**AMY/HPMC 4M**	130	135	155	165	70–50	0.8–0.3	60–40	+/−	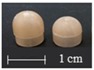

* processability: −, incomplete/broken/deformed units; −/+, complete unit manually removed/extremely adhesive; +, complete unit, occasionally automatically ejected; ++, +, complete unit, automatically ejected.

## Abbreviations

AMY	Amylo^®^ N-460
CFU	Colony-forming unit
3D	Three-dimensional
DDSs	Drug delivery systems
GLY	Glycerol
HME	Hot melt extrusion
HPC	Hydroxypropyl cellulose
HPMC	Hydroxypropyl methylcellulose
HPLC	High pressure liquid chromatography
IM	Injection molding
LYC	Lycoat^®^ RS780
PEG	Polyethylene glycol
PVA	Poly(vinyl alcohol)
RDM	Residual dry mass
USP	United States Pharmacopeia
WC	Water content
